# The December No. of the Journal for 1867

**Published:** 1867-12

**Authors:** 


### Editorial.

### *The December No. of the Journal, 1867,*

Has been delayed a few days *necessarily*, because of change in the periods of publication intended for the ensuing year.

The present Editor took charge of the JOURNAL in March last, when its prospects were not singularly flattering. During the year, he has been under the pressure of other engagements of the most urgent character, which have prevented him from paying that attention to the JOURNAL which it really merited. Nevertheless, its subscription list has been nearly doubled, and assurances of support of the most gratifying character have been freely tendered.

The treatise on the ENDOSCOPE, not otherwise to be procured, has been afforded to subscribers complete, notwithstanding the unfortunate demise of our friend, R. P. Hunt, M.D., who first undertook its translation.

On looking over the numbers, our friends will see that we have from time to time issued supplementary "forms," and indeed the present No. presents seventy-two instead of forty-eight pages, the usual *quantum*.

What we feel to have been lacking the last year is *time*, of which the Editor had little to spare.

The ensuing year, arrangements have been made which will enable the Editor to devote more time to preparation of the JOURNAL, and such assistance has been secured as can not fail to make its contents of a useful and instructive character.

Foreign correspondence, local news, and original articles of valuable character will be sandwiched with Editorial comments and reflections. Suffice it to say, we intend to make the JOURNAL a necessity for medical gentlemen in the North-west.

